# An integrated analysis of bulk and single-cell sequencing data reveals that EMP1^+^/COL3A1^+^ fibroblasts contribute to the bone metastasis process in breast, prostate, and renal cancers

**DOI:** 10.3389/fimmu.2023.1313536

**Published:** 2023-12-19

**Authors:** Haoyuan Du, Hua Wang, Yuwei Luo, Yang Jiao, Jiajun Wu, Shaowei Dong, Dong Du

**Affiliations:** ^1^ Department of Orthopedics and Joints, Shenzhen People’s Hospital (The Second Clinical Medical College, Jinan University, The First Affiliated Hospital, Southern University of Science and Technology), Shenzhen, Guangdong, China; ^2^ Department of Breast Surgery, Shenzhen People’s Hospital (The Second Clinical Medical College, Jinan University; The First Affiliated Hospital, Southern University of Science and Technology), Shenzhen, Guangdong, China; ^3^ Department of Ultrasound, Shenzhen People’s Hospital (The Second Clinical Medical College, Jinan University, The First Affiliated Hospital, Southern University of Science and Technology), Shenzhen, Guangdong, China; ^4^ Department of Pediatric Research, Shenzhen Children’s Hospital, Shenzhen, Guangdong, China; ^5^ Department of Health Management, Shenzhen People’s Hospital (The Second Clinical Medical College, Jinan University, The First Affiliated Hospital, Southern University of Science and Technology), Shenzhen, Guangdong, China

**Keywords:** EMP1+/COL3A1+ fibroblasts, bone metastasis, scRNAseq, multi-tumor TMEs, combined analysis

## Abstract

**Introduction:**

Bone metastasis (BoM) occurs when cancer cells spread from their primary sites to a bone. Currently, the mechanism underlying this metastasis process remains unclear.

**Methods:**

In this project, through an integrated analysis of bulk-sequencing and single-cell RNA transcriptomic data, we explored the BoM-related features in tumor microenvironments of different tumors.

**Results:**

We first identified 34 up-regulated genes during the BoM process in breast cancer, and further explored their expression status among different components in the tumor microenvironment (TME) of BoM samples. Enriched EMP1+ fibroblasts were found in BoM samples, and a COL3A1-ADGRG1 communication between these fibroblasts and cancer cells was identified which might facilitate the BoM process. Moreover, a significant correlation between EMP1 and COL3A1 was identified in these fibroblasts, confirming the potential connection of these genes during the BoM process. Furthermore, the existence of these EMP1+/COL3A1+ fibroblasts was also verified in prostate cancer and renal cancer BoM samples, suggesting the importance of these fibroblasts from a pan-cancer perspective.

**Discussion:**

This study is the first attempt to investigate the relationship between fibroblasts and BoM process across multi-tumor TMEs. Our findings contribute another perspective in the exploration of BoM mechanism while providing some potential targets for future treatments of tumor metastasis.

## Introduction

Primary bone cancer refers to cancer that develops from cancerous bone progenitor cells, which is rare and accounts for less than 1% of all cancers; metastatic bone cancer or secondary bone cancer refers to cancer that originates from other sites and spreads to bones. Due to the rich arterial supply, solid tumors frequently metastasize to bone (1, 2). Among all primary cancer types, prostate cancer has the highest probability (34%) of bone metastasis (BoM, hereafter), followed by breast cancer (22%) and lung cancer (20%) (3). BoM could greatly reduce patients’ survival in many different cancers, such as prostate cancer (at least 12 months), breast cancer (at least 19 months), and lung cancer (at least 6 months) (4–6).

During the past several decades, tremendous effort has been performed in deciphering the BoM mechanism (7, 8). Generally, BoM involves several steps including cancer cell escape and dissemination, cancer cell invasion, and metastasis formation in the bone (9). The interactions between receptors on cancer cells such as CXCR4 (10, 11) and RANKL (12) and ligands on stroma cells such as CXCL12 (13) was considered as one of the important pathogenesis mechanism of BoM. These invaded cancer cells further release cytokines such as IL6 (14) and angiogenic factors including VEGF (15, 16) to promote tumor progression in the new sites. Due to their high heterogeneity, currently, it is difficult to explore the commonalities of the BoM mechanism pan-cancer-wide.

Bone marrow is a multi-cell-type containing system, and many of these components could promote the BoM process, such as hematopoietic progenitor cells (HPCs) (17), mesenchymal stem cells (MSCs) (16), osteocytes (18), etc. Recently, with the advancement of single-cell RNA sequencing (scRNAseq) technologies, it is now possible to explore the BoM process from single-cell levels while examining significantly changed communications among different TME components (19). In this study, using an integrated analysis on bulk and scRNAseq transcriptomic datasets, we provide evidence that EMP1^+^/COL3A1^+^ fibroblasts are enriched in BoM samples of breast, prostate, and renal cancers, and these fibroblasts might contribute to the BoM process through potential COL3A1-ADGRG1 communication with cancer cells.

## Materials and methods

### Bulk sequencing data retrieval

The phenotypic data of 114,311 TCGA (The Cancer Genome Atlas Program) samples referring to 33 tumors were retrieved from the GDC portal (Genomic Data Commons data portal, www.portal.gdc.cancer.gov). Tumor samples were categorized using “sample_type.samples” information (“Primary Tumor”); meta samples were determined using “metastatic_site” information; BoM samples were determined using “new_neoplasm_occurrence_anatomic_site_text” information (with “bone” letters in all sites); gene expression data (counts) of breast cancer (BRCA) was also downloaded from the GDC portal. ACC: adrenocortical carcinoma; BLCA: bladder urothelial carcinoma; BRCA: breast invasive carcinoma; CESC: cervical squamous cell carcinoma and endocervical adenocarcinoma; CHOL: cholangiocarcinoma; COAD: colon adenocarcinoma; DLBC: lymphoid neoplasm diffuse large B-cell lymphoma; ESCA: esophageal carcinoma; GBM: glioblastoma multiforme; HNSC: head and neck squamous cell carcinoma; KICH: kidney chromophobe; KIRC: kidney renal clear cell carcinoma; KIRP: kidney renal papillary cell carcinoma; LAML: acute myeloid leukemia; LGG: brain lower grade glioma; LIHC: liver hepatocellular carcinoma; LUAD: lung adenocarcinoma; LUSC: lung squamous cell carcinoma; MESO: mesothelioma; OV: ovarian serous cystadenocarcinoma; PAAD: pancreatic adenocarcinoma; PCPG: pheochromocytoma and paraganglioma; PRAD: prostate adenocarcinoma; READ: rectum adenocarcinoma; SARC: sarcoma; SKCM: skin cutaneous melanoma; STAD: stomach adenocarcinoma; TGCT: testicular germ cell tumors; THCA: thyroid carcinoma; THYM: thymoma; UCEC: uterine corpus endometrial carcinoma; UCS: uterine carcinosarcoma; UVM: uveal melanoma.

### Differential gene expression analysis

Deseq2 (R package) was used in the differential gene expression analysis. Raw-count reads of involved samples from the TCGA database were used as input. The adjusted p-value of 0.05 was used as a significance cutoff.

### Enrichment analysis

Gene Ontology (GO) and Kyoto Encyclopedia of Genes and Genomes (KEGG) enrichment analysis was performed using the R package “clusterProfiler” and “org.Hs.eg.db” database. “ENTREZID” was used in the analysis. Adjusted p-values were achieved using a “Benjamini-Hochberg” adjustment method. The adjusted p-value of 0.05 was used as a significance cutoff.

### Single-cell RNA sequencing data analysis

Three sets of scRNAseq data were used in this study: breast cancer data GSE190772 (20), prostate cancer GSE143791 (21), and renal cancer GSE202813. Count matrix files were retrieved from the Gene Expression Omnibus (GEO, www.ncbi.nlm.nih.gov/geo/) database. R “Seurat” package (Version 3.12) was used to construct SeuratObjects using count matrix files (22); R “DoubletFinder” package was used to perform doublet removal process (23); and R “Seurat” package “Findintegrationanchors” and “integrateData” functions were used to perform integration process. The Uniform Manifold Approximation and Projection (UMAP) method was used to perform the non-linear dimensional reduction process.

### Ligand-receptor analysis

The L-R interactions between subgroup A (Ligands) and subgroup B (Receptors) were screened using the following steps: (1) All L-R pair information was downloaded from the cellphoneDB database (24); (2) All cells from subgroup A were separated into two groups: cells derived from BoM samples (BoM) and cells derived from other samples (Other), and a Wilcoxon rank-sum comparison was used to compare expression differences of all ligand genes between these two groups, and only significantly upregulated ligands in BoM cells were selected (p-value < 0.05); (3) For all the significantly upregulated ligands in BoM cells, only ones with positive expression ratio over 10% (the number of cells with positive expression scores among BoM cells divided by all the number of BoM cells) were selected; (4) Receptor candidates in subgroup B were screened using step (2) and step (3); (5) The L-R pairs formed by ligand candidates from subgroup A and receptor candidates from subgroup B were considered as L-R candidate interactions.

### Correlation analysis

All the correlation analysis involved in this study was performed using the R cor.test() function, and “Pearson” method was applied in each analysis. A p-value of 0.05 was used as a significance cutoff.

## Results

### Project design

**Figure 1:**
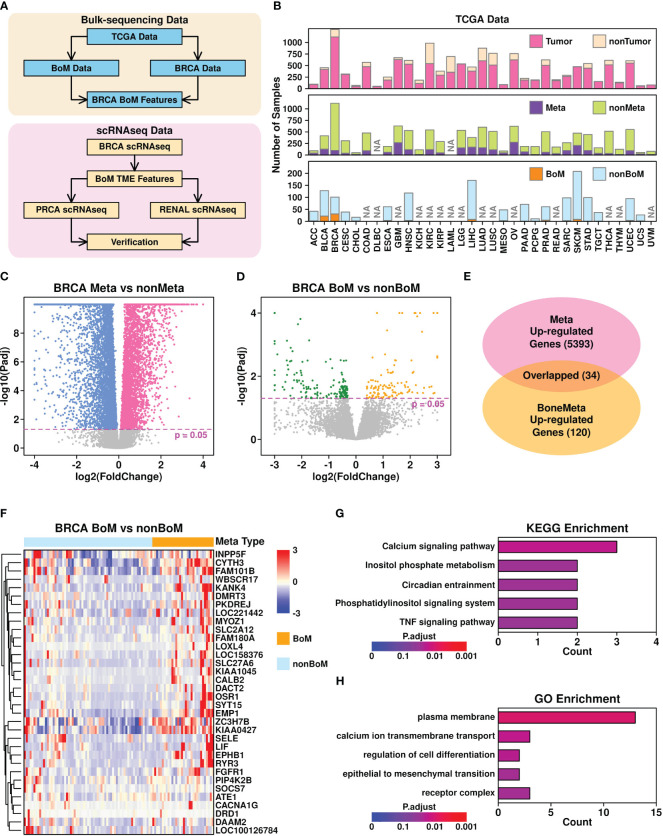
Bone metastasis-related features revealed in bulk-sequencing data. **(A)** Flowchart describing the research design of this study; **(B)** Summary of sample information from TCGA database. NA, no records; **(C)** Volcano plot illustrating the identification of differentially expressed genes in Meta samples (samples with metastasis records) vs non-Meta samples (samples without metastasis records); **(D)** Volcano plot illustrating the identification of differentially expression genes in BoM samples (metastasis samples with bone metastasis records) vs nonBoM samples (metastasis samples without bone metastasis records); **(E)** Overlap between upregulated genes in Meta samples and BoM samples; **(F)** Expression status of upregulated genes in Meta samples; **(G)** Boxplot representing the KEGG enrichment results of 34 upregulated genes; **(H)** Boxplot representing the GO enrichment results of 34 upregulated genes.

The project design of this study is illustrated in **Figure 1A**. In this study, we first compared transcriptome level gene expression data between TCGA BoM samples and other samples using datasets from the TCGA database and screened a 34-gene panel that is highly expressed in BRCA BoM samples. The tumor is a complicated system containing various components, including tumor cells, fibroblasts, immune cells, etc., however, bulk-sequencing data could only represent homogenized expression data across the whole tissue, hence these upregulated genes could not reflect the key tumor environmental features relating to bone metastasis. Using scRNAseq data, we further explored the expressional status of these 34 genes in breast cancer BoM samples from a TME perspective, identified a BoM-related component, and further verified the existence of this component in BoM samples of two other cancers (prostate cancer and renal cancer). These two cancers plus breast cancer are the only three cancers with BoM scRNAseq data that can be downloaded from online databases.

### Identification of 34 upregulated genes in breast cancer BoM samples

To explore the BoM-related features pan-cancer-wide, we first retrieved the phenotypic data referring to 33 cancers from the TCGA online database, and summarized their status, as shown in **Figure 1B** and **Supplementary Table 1**. Among all different cancer types, 18 of them have tumor samples with BoM records and only 2 of them have more than 10 samples with BoM records: breast invasive carcinoma (BRCA) with 32 samples and bladder urothelial carcinoma (BLCA) with 22 samples. Hence in the following analysis, we used BRCA data to explore BoM-related gene features.

Through Deseq2 (25) analysis, we first identified 5,393 upregulated genes (adjusted p-value < 0.05) in BRCA samples with metastasis records (Meta) compared to those without metastasis records (non-Meta), as shown in **Figure 1C**. We further identified 120 upregulated genes in BRCA samples with BoM records compared to these metastasis samples without BoM records (non-BoM), as shown in **Figure 1D**. A total of 34 overlapped genes were found (**Figure 1E**) and their expression status among metastasis samples is shown in **Figure 1F**. The top 5 Kyoto Encyclopedia of Genes and Genomes (KEGG) enriched terms of these 34 genes include “calcium signaling pathway”, “inositol phosphate metabolism”, “circadian entrainment”, “phosphatidylinositol signaling system”, and “TNF signaling pathway”, as shown in **Figure 1G**. The top 5 Gene Ontology (GO) enriched terms include “plasma membrane”, “calcium ion transmembrane transport”, “regulation of cell differentiation”, “epithelial to mesenchymal transition”, and “receptor complex”, as shown in **Figure 1H**.

### BoM-related tumor microenvironment features in breast cancer

**Figure 2:**
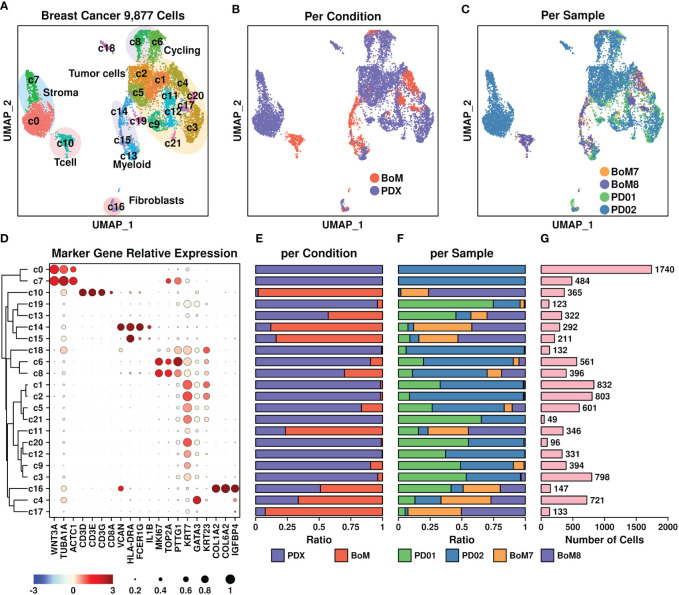
Breast cancer scRNAseq data. **(A)** UMAP plot showing the distribution patterns of all subgroups; **(B)** UMAP plot showing the distribution patterns of all subgroups per sample type; **(C)** UMAP plot showing the distribution patterns of all subgroups per sample; **(D)** Dot plot representing the relative expression status of all marker genes used in the determination of each subgroup. The relative expression levels of each gene are represented in different colors (red: high; blue: low. Z-score normalization is performed). The ratios of cells with expressions of specific genes (count > 0) are represented by the size of the circle; **(E–G)** Bar plots showing the basic information of all 22 subgroups including ratio per condition **(E)**, ratio per sample **(F)**, and number of cells **(G)**.

To investigate the expressional status of these upregulated genes from cellular levels, we retrieved breast cancer BoM scRNAseq data from the GEO database under accession GSE190772 (20) and reconstructed the UMAP plot representing the distributions of 9,877 cells based on representative markers (**Figure 2A**). Four samples were included in this dataset: two PDX samples (PD01 and PD02) and two BoM samples (BoM7, BoM8). The UMAP plots are shown in **Figure 2B** (per condition) and (**Figure 2C**) (per sample).

All subgroups were further clustered into the following groups (**Figure 2D**): T cells (Tcell) including c10 based on CD3D/CD3E/CD3G genes, myeloids including c14 and c15 based on VCAN/HLA-DRA/FCGR1G genes, stroma cells including c7 and c0 based on WNT3A/TUBA1A/ACTC1 genes, fibroblasts including c16 based on COL1A2/COL6A2/IGFBP4 genes, cycling cells including c6 and c8 based on TOP2A/MKI67/PTTG1 genes, and tumor cells including c1, c2, c5, c21, c11, c20, c12, c9, and c3 based on KRT7/GATA3/KRT23 genes. The ratios representing cells from different origins including conditions and samples, as well as the number of cells in each subgroup, are illustrated in **Figures 2E–G**, respectively. Among these subgroups, most of the T cells (c10) and myeloids (c14, c15) were derived from BoM samples, suggesting a different tumor immune microenvironment between PDX and BoM samples. Regarding tumor cells, higher proportions of c4, c11, and c17 cells were derived from BoM samples, suggesting a potential relationship between these cells and the BoM process.

**Figure 3:**
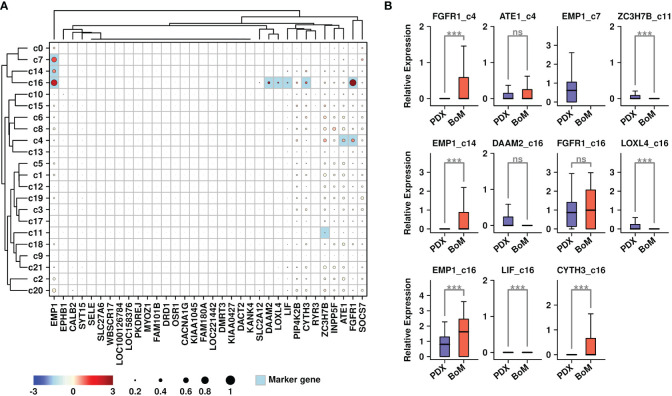
Expression status of BoM-featured genes. **(A)** Expression status of 34 upregulated genes among all subgroups. Overlapped genes between 34 upregulated genes and subgroup marker genes were highlighted in light blue color. The relative expression levels of each gene are represented in different colors (red: high; blue: low. Z-score normalization is performed); The ratios of cells with expressions of specific genes (count > 0) are represented by the size of the circle; **(B)** Expression status of target genes among candidate subgroups. A Wilcoxon rank-sum test was performed. ***: p < 0.001.

We further explored the expressional status of 34 upregulated genes among different subgroups, and the results are shown in **Figure 3A**. The marker genes from each subgroup were also calculated using the R Seurat FindAllMarkers() function. The overlapped candidates between these 34 genes and marker genes from each subgroup were highlighted in light blue color. Among different subgroups, c16 cells (fibroblasts) had higher expression of six genes (EMP1/DAAM2/LOXL4/LIF/CYTH3/FGFR1); c4 (tumor cells) had higher expression of two genes (ATE1/FGFR1); c7 (stroma cells) and c14 (myeloid) had higher expression of one gene (EMP1); and c11 (tumor cells) had higher expression of one gene (ZC3H7B). To investigate whether these genes might be related to bone metastasis, we compared the expression levels of these gene-subgroup combinations between cells from PDX samples and BoM samples (**Figure 3B**). Among all combinations, there were higher expressions of FGFR1 in BoM c4 cells, EMP1 in BoM c14 cells, and EMP1/CYTH3 in BoM c16 cells compared to these genes in cells from PDX samples, implying the possible relationship of these gene-subgroup combinations to the BoM process.

### Involvement of EMP1^+^ Fibroblasts during the bone metastasis process in breast cancer

**Figure 4:**
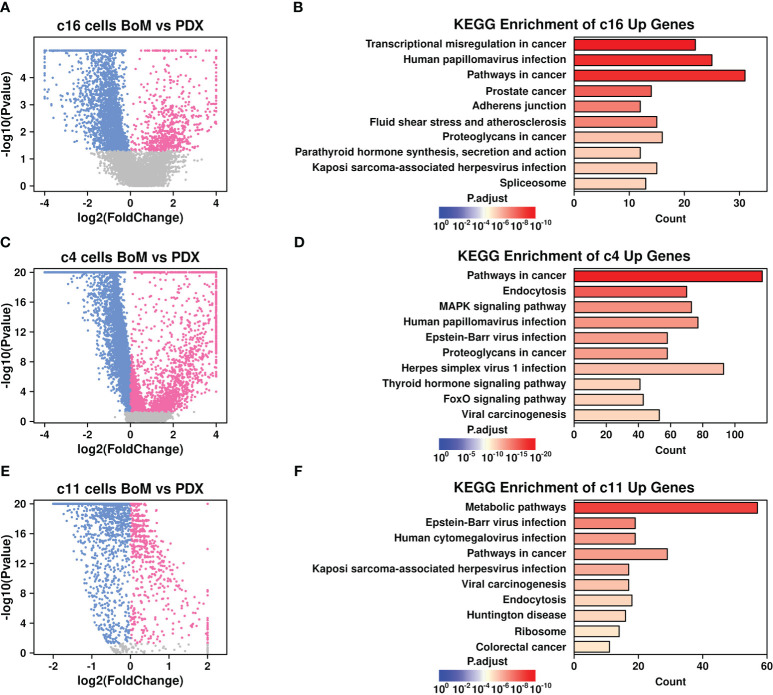
Gene expression features in BoM-derived subgroups. Volcano plots showing differentially expressed genes in c16 subgroup **(A)**, c4 subgroup **(C)**, and c11 subgroup **(E)**. Bar plots showing top enriched KEGG items of upregulated genes from BoM-derived cells in c16 subgroup **(B)**, c4 subgroup **(D)**, and c11 subgroup **(F)**.

To explore the specific gene features that might facilitate the BoM process in breast cancer, we further screened for upregulated genes in BoM cells among these subgroups. Only genes with expression values in more than 10% of the cells were involved in the screening, and a Wilcoxon-rank sum test was performed during the comparison. There are 723 upregulated genes in BoM-derived c16 cells (**Figure 4A** and **Supplementary Table 2**), and the top 10 enriched KEGG terms are listed in **Figure 4B**. There were 2,867 upregulated genes in BoM-derived c4 cells (**Figure 4C** and **Supplementary Table 3**), and the top 10 enriched KEGG terms are listed in **Figure 4D**. There were 569 upregulated genes in BoM-derived c11 cells (**Figure 4E** and **Supplementary Table 4**), and the top 10 enriched KEGG terms are listed in **Figure 4F**. Most of the upregulated genes in BoM-derived cells were enriched in cancer-related pathways (including “transcriptional misregulation in cancer”, “pathways in cancer”, “proteoglycans in cancer”, etc.), confirming the importance of these cells during tumor progression.

**Figure 5:**
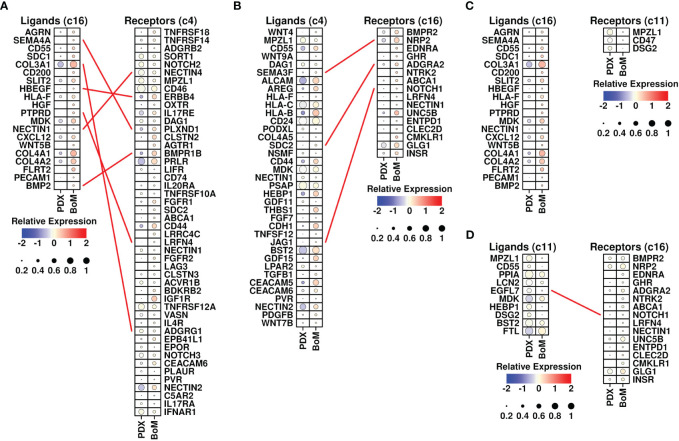
Ligand-receptor analysis. **(A)** L-R interactions between ligands from the c16 subgroup (upregulated in BoM-derived cells) and receptors from the c4 subgroup (upregulated in BoM-derived cells); **(B)** L-R interactions between ligands from the c4 subgroup (upregulated in BoM-derived cells) and receptors from the c16 subgroup (upregulated in BoM-derived cells); **(C)** L-R interactions between ligands from the c16 subgroup (upregulated in BoM-derived cells) and receptors from the c11 subgroup (upregulated in BoM-derived cells); **(D)** L-R interactions between ligands from the c11 subgroup (upregulated in BoM-derived cells) and receptors from the c16 subgroup (upregulated in BoM-derived cells); The relative expression levels of each gene are represented in different colors (red: high; blue: low. Z-score normalization is performed). The ratios of cells with expressions of specific genes (count > 0) are represented by the size of the circle.

Cancer-associated fibroblasts (CAFs) are fibroblasts that are enriched in tumor sites, and the involvement of CAFs during the BoM process has been revealed in many studies (26). To investigate the specific communications that might aid the BoM process between fibroblasts and tumor cells, we screened the ligand-receptor (L-R) combinations that were both upregulated in BoM-derived c16/c4/c11 cells (detailed in Materials and Methods) and summarized their communications (**Figure 5**). There were five L-R interactions between c16 (as ligands) and c4 (as receptors) subgroups, namely, SEMA4A-PLXND1, COL3A1-ADGRG1, HBEGF-ERBB4, PTPRD-ADGRG1, and BMP2-BMPR1B, and three interactions between c4 (as ligands) and c16 (as receptors) subgroups, namely, SEMA3F-BMPR2, SDC2-ADGRA2, and JAG1-NOTCH1. There were zero interactions between c16 (as ligands) and c11 (as receptors) subgroups, and one interaction between c11 (as ligands) and c16 (as receptors) subgroups, namely, EGFL7-NOTCH1. More communications were found between the c4 and c16 subgroups, suggesting that the communications between these two subgroups might play more important roles during the BoM process.

Among invaded tumor cells, the c4 subgroup had elevated expression of TGFB1 (**Figure 5B**). The involvement of TGF-beta in the BoM process has been shown in many studies, as summarized in Trivedi et al. (27) TGF-beta could promote both the growth of tumor cells and the epithelial mesenchymal process, as well as suppress the immune responses (28). Among c16 fibroblasts, there were increased expressions of CXCL12 and BMP2. CXCL12 secreted by stroma cells plays a pivotal role in bone metastasis (13), and blocking the CXCL12/CXCR4 axis significantly inhibited the BoM process in prostate cancer (29). BMP2 has also been shown to be involved in the BoM process among many different cancers including lung carcinoma (30) and breast cancer (31). All these cytokines secreted by invading cancer cells and fibroblasts could facilitate the BoM process and need further exploration.

**Figure 6:**
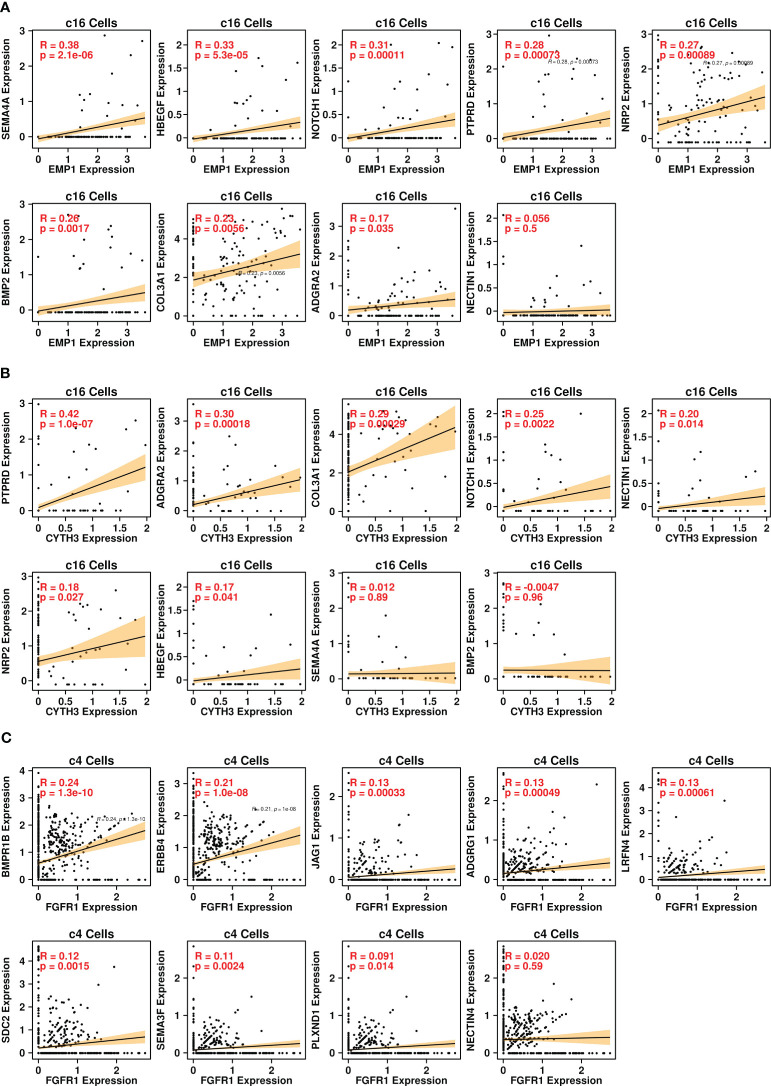
Pearson correlation analysis. **(A)** correlations between expression levels of EMP1 and ligand genes in the c16 subgroup; **(B)** correlations between expression levels of CYTH3 and ligand genes in the c16 subgroup; **(C)** correlations between expression levels of EMP1 and receptor genes in the c4 subgroup.

Through Pearson analysis, we also examined significant correlations between EMP1/CYTH3 genes and L-R-related genes in c16 cells, as shown in **Figures 6A, B**. High correlation results (R > 0.2) were found between the expression levels of SEMA4A/HBEGF/NOTCH1/PTPRD/NRP2/BMP2/COL3A1 and EMP1 genes in c16 cells, and high correlation (R > 0.2) was found between the expression levels of PTPRD/ADGRA2/COL3A1/NOTCH1 and CYTH3 genes in c16 cells. The correlations between the FGFR1 gene and other L-R-related genes in c4 cells were also examined, as shown in **Figure 6C**. High correlation (R > 0.2) results were found between BMPR1B/ERBB4 genes and FGFR1 genes in c4 cells. All these results suggest that these BoM-related genes might function through the communications between fibroblasts and tumor cells.

### BoM-related tumor microenvironment features in prostate cancer

**Figure 7:**
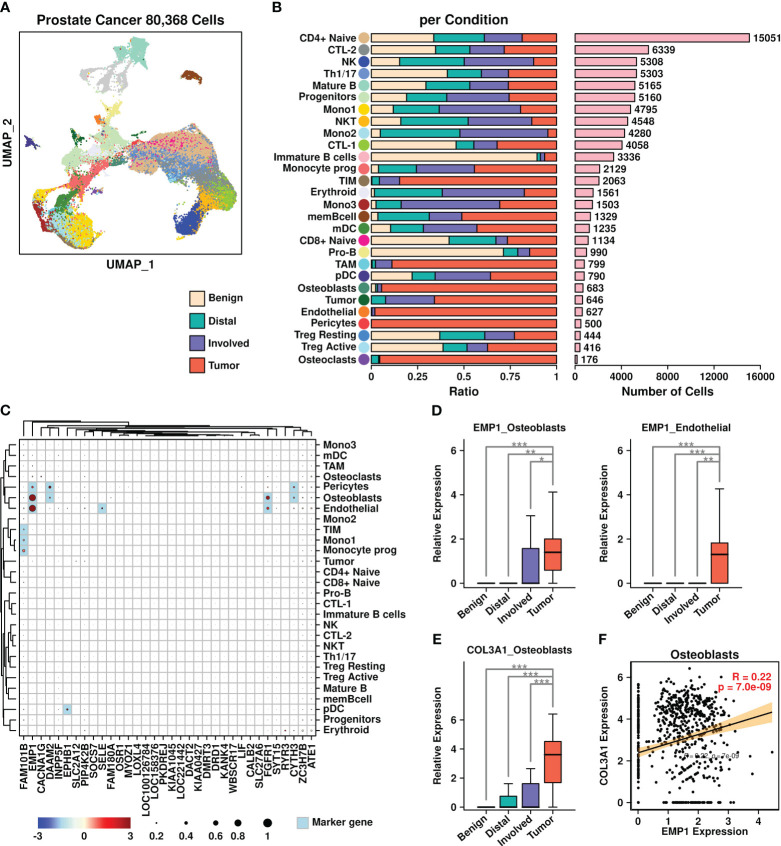
Prostate cancer scRNAseq data analysis. **(A)** UMAP plot showing the distribution patterns of all subgroups; **(B)** Bar plots showing the basic information of all 28 subgroups including ratio per condition and the number of cells; **(C)** Expression status of 34 upregulated genes among all subgroups. Overlapped genes between 34 upregulated genes and subgroup marker genes were highlighted in light blue color. The relative expression levels of each gene are represented in different colors (red: high; blue: low. Z-score normalization is performed). The ratios of cells with expressions of specific genes (count > 0) are represented by the size of the circle; **(D)** Expression EMP1 among different sample types in osteoblasts and endothelial cells; **(E)** Expression COL3A1 among different sample types in osteoblasts. A Wilcoxon rank-sum test was performed. *: p < 0.05; **: p < 0.01; ***: p < 0.001, and a “Bonferroni” correction was performed for multiple testing; **(F)** Pearson correlation between expression levels of EMP1 and COL3A1 in osteoblasts.

To investigate the expression status of these upregulated genes in prostate cancer BoM samples, we retrieved PRAD BoM scRNAseq data from the GEO database (GSE143791) and reconstructed a UMAP plot (**Figure 7A**) representing the distributions of 80,368 cells based on the annotations from the original publication (21). There are four types of samples involved in this dataset: benign bone marrow controls (Benign), liquid bone marrow distant from the tumor site (Distal), liquid bone marrow in the spinal cord (Involved), and solid metastatic tissue (Tumor). The proportions of cells derived from each sample type among different subgroups are depicted in **Figure 7B**, with the number of cells listed in **Figure 7C**.

We further examined the expression status of 34 BoM metastasis-related genes among different subgroups (**Figure 7C**). Among different subgroups, osteoblasts had higher expression of four genes (EMP1/DAAM2/FGFR1/CYTH3); pericytes had higher expression of three genes (EMP1/DAAM2/CYTH3); endothelial cells had higher expression of three genes (EMP1/SELE/FGFR1); tumor inflammatory monocytes (TIM) cells, monocytes subgroup 1 (Mono1) cells and Monocyte prog (progenitor) cells had higher expression of one gene (FAM101B); plasmacytoid dendritic cells (pDC) cells had higher expression of one gene (EPHB1). Among these gene/subgroup combinations, there was increased expression of EMP1 in tumor cells compared to other cells in osteoblasts and endothelial cells. Among all LR communication partners that were screened in BRCA BoM datasets, only COL3A1 had higher expression in osteoblasts derived from tumor samples compared to osteoblasts derived from other samples (**Figure 7E**). Furthermore, there was a significant correlation between COL3A1 and EMP1 in osteoblasts (**Figure 7F**), suggesting the enrichment of COL3A1^+^/EMP1^+^ cells during the BoM process in prostate cancer.

### BoM-related tumor microenvironment features in renal cancer

**Figure 8:**
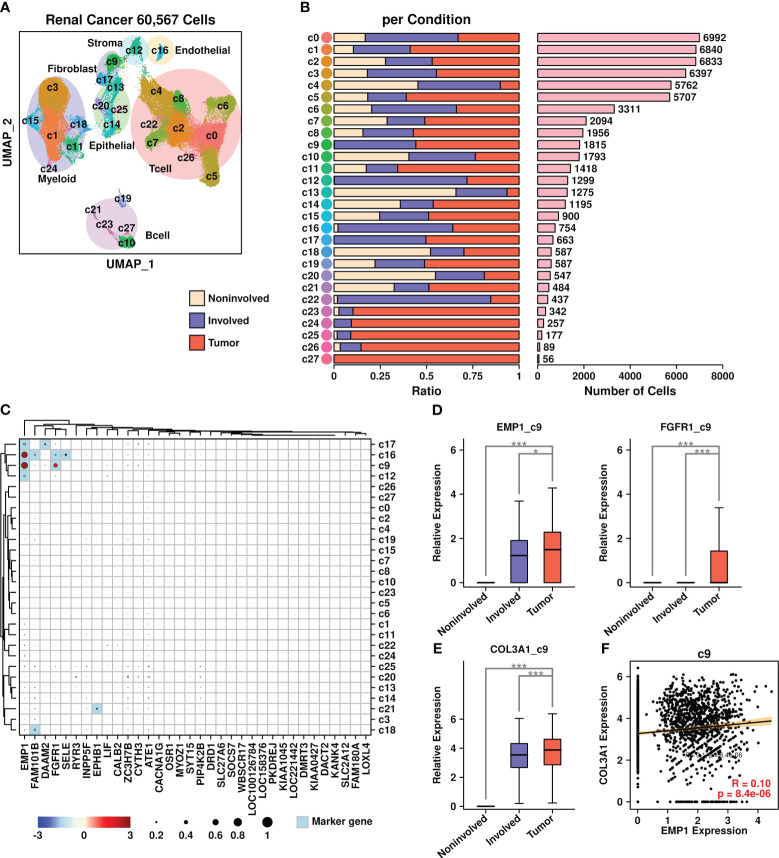
Renal cancer scRNAseq data analysis. **(A)** UMAP plot showing the distribution patterns of all subgroups; **(B)** Bar plots showing the basic information of all 28 subgroups including ratio per condition and the number of cells; **(C)** Expression status of 34 upregulated genes among all subgroups. Overlapped genes between 34 upregulated genes and subgroup marker genes were highlighted in light blue color. The relative expression levels of each gene are represented in different colors (red: high; blue: low. Z-score normalization is performed). The ratios of cells with expressions of specific genes (count > 0) are represented by the size of the circle; **(D)** Expression EMP1 among different sample types in the c9 subgroup; **(E)** Expression COL3A1 among different sample types in the c9 subgroup. A Wilcoxon rank-sum test was performed. *: p < 0.05; **: p < 0.01; ***: p < 0.001, and a “Bonferroni” correction was performed for multiple testing; **(F)** Pearson correlation between expression levels of EMP1 and COL3A1 in the c9 subgroup.

We further verified the existence of COL3A1^+^/EMP1^+^ cells in renal cancer BoM samples, as shown in **Figure 8**. Renal cell BoM scRNAseq data from the GEO database (GSE202813) was retrieved and a UMAP plot was constructed (**Figure 8A**). There were three types of samples involved in this dataset: liquid bone marrow distant from the tumor site used as control (Non-involved), liquid bone marrow in the spinal cord (Involved), and solid metastatic tissue (Tumor). The proportions of cells derived from each sample type among different subgroups are depicted in **Figure 8B**, with the number of cells listed in **Figure 8C**.

The expression status of 34 BoM metastasis-related genes among different subgroups was analyzed and is shown in **Figure 8C**. Among different subgroups, c16 cells (endothelial cells) had higher expression of four genes (EMP1/FAM101B/FGFR1/SELE); c9 cells (fibroblasts) had higher expression of two genes (EMP1/FGFR1); c17 cells (fibroblasts) had higher expression of two genes (EMP1/DAAM2); c12 cells (stroma cells) had higher expression of one gene (EMP1); c21 cells (B cells) had higher expression of one gene (EPHB1); c18 cells (myeloid) had higher expression of one gene (FAM101B). Among these gene/subgroup combinations, there was increased expression of EMP1 and FGFR1 in tumor cells compared to other cells in c9 fibroblasts. Among all LR communication partners that were screened in BRCA BoM datasets, only COL3A1 had higher expression in c9 fibroblasts derived from tumor samples compared to c9 fibroblasts derived from other samples (**Figure 8E**). Furthermore, there was a significant correlation between COL3A1 and EMP1 in c9 fibroblasts (**Figure 8F**), confirming the existence of COL3A1^+^/EMP1^+^ cells during the bone metastasis process in renal cell carcinoma.

### Correlation between EMP1/COL3A1 and epidermal mesenchymal transition process

**Figure 9:**
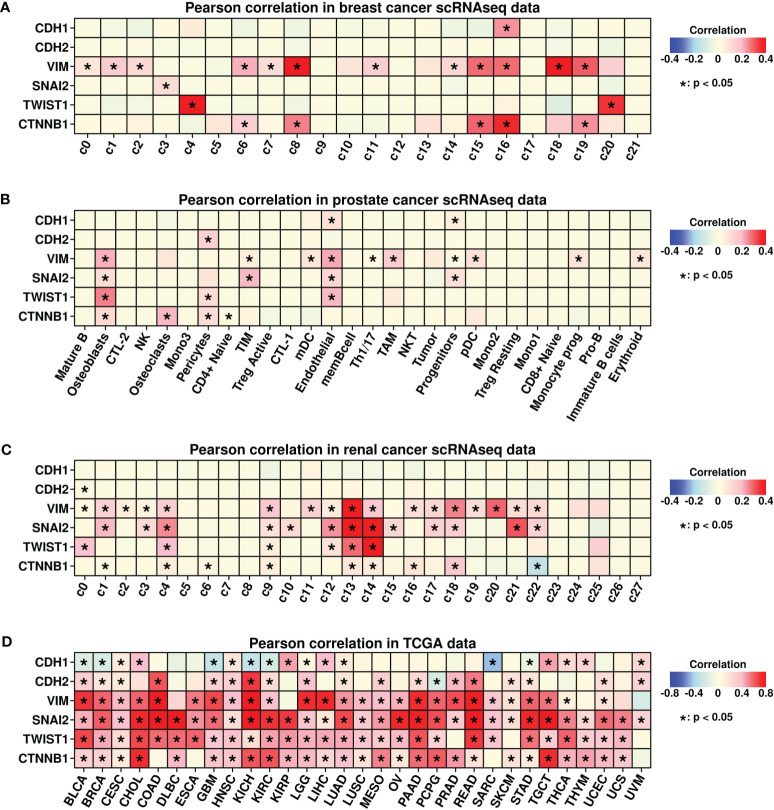
Pearson correlation analysis. **(A)** Pearson correlation analysis between expression levels of COL3A1/EMP1 genes and EMT-related genes in different subgroups of breast cancer scRNAseq data; **(B)** Pearson correlation analysis between expression levels of COL3A1/EMP1 genes and EMT-related genes in different subgroups of prostate cancer scRNAseq data; **(C)** Pearson correlation analysis between expression levels of COL3A1/EMP1 genes and EMT-related genes in different subgroups of renal cancer scRNAseq data; **(D)** Pearson correlation analysis between expression levels of COL3A1/EMP1 genes and EMT-related genes in bulk-sequencing data of 28 different cancers from the TCGA database; Z-score normalization was performed to get the expression data of COL3A1/EMP1 genes.

Epidermal mesenchymal transition (EMT) is a cellular process involving epithelial cells acquiring mesenchymal features (32) and is considered an important step during tumor metastasis (33). In the present study, we further explored the correlation status between EMP1/COL3A1 and EMT-related proteins including E-cadherins (CDH1), N-Cadherins (CDH2), Vimentin (VIM), Slug (SNAI2), Twist (TWIST1), and beat catenin (CTNNB1). Among different subgroups in breast cancer scRNAseq data (**Figure 9A**), significant correlations between EMP1/COL3A1 and three EMT genes (CDH1, VIM, and CTNNB1) were found in c16 fibroblasts. Among different subgroups in prostate cancer scRNAseq data (**Figure 9B**), significant correlations between EMP1/COL3A1 and four EMT genes (VIM, SNAI2, TWIST1, and CTNNB1) were found in the osteoblast subgroup. Among different subgroups in renal cancer scRNAseq data (**Figure 9C**), significant correlations between EMP1/COL3A1 and four EMT genes (VIM, SNAI2, TWIST1, and CTNNB1) were found in c9 fibroblasts.

We also explored the correlations between EMP1/COL3A1 and EMT genes pan-cancer-wide using TCGA data, and the results are shown in **Figure 9D**. Significant correlations were found in most of the cancer types. All these findings confirm the importance of EMP1/COL3A1 during the EMT process while implying that the mechanism underlying BoM promoting EMP1+/COL3A1+ fibroblasts could be explored through the perspective of EMT.

## Discussion

Tumor metastasis is a complicated process involving multiple steps: (1) the survival and proliferation of cancer cells at primary sites; (2) the invasion of cancer cells to adjacent tissues; (3) extravasation into blood or lymph nodes to reach distant organs; (4) the survival and proliferation of these cells at metastatic sites after seeded in distant organs (34, 35). The involvement of CAFs in all these steps has been verified in many studies, as summarized in Gascard and Tlsty (36) and Bu et al. (37) During the BoM process, Zhang et al. (38) found that CAFs in breast cancer could produce CXCL12 to screen for Src^High^ tumor cells, and these tumor cells could be further attracted to CXCL12^High^ bone marrows to facilitate the BoM process. Shahriari et al. (39) discovered that in prostate cancer, IL1B^+^ cancer cells could cooperate with S100A4^+^ CAFs to promote the BoM process. In our results, we also found the involvement of EMP1^+^/COL3A1^+^ fibroblasts during the BoM process in breast and renal cancer, which is consistent with previous findings. In prostate cancer, more EMP1^+^/COL3A1^+^ osteoblasts were found instead of fibroblasts. Since osteoblasts are generally considered as one type of fibroblasts (40) and the conversion of fibroblasts to osteoblasts has been examined in multiple studies (41, 42), enrichment of EMP1^+^/COL3A1^+^ osteoblasts in BoM samples of prostate cancer also demonstrates the importance of fibroblasts during the BoM process.

The epithelial membrane protein (EMP) family includes three members: EMP1, EMP2, and EMP3. Amin et al. (43) found that elevated EMP1 expression could significantly promote the invasion process of tumor cells into lymph nodes and lungs in prostate cancer, indicating the potential role of EMP1 in the BoM process. In this study, we first identified EMP1 as one of the 34 upregulated genes in BoM samples compared to non-BoM samples in TCGA BRCA datasets (**Figure 1F**). Furthermore, we found enhanced expression of EMP1 in BoM-derived fibroblasts in breast cancer (**Figure 3B**), and these cells were enriched in multiple cancer-related pathways (**Figure 4B**), confirming the importance of these cells during the BoM process. Through L-R analysis, we identified COL3A1-ADGRG1 as a potential communication interaction between these fibroblasts and cancer cells (**Figure 5A**). The involvement of ADGRG1, one of the G-protein coupled receptors, in tumor progression has been reported in many studies, as summarized in Ng et al. (44). Recently, Sasaki et al. (45) found that suppression of ADGRG1 in breast cancer cells could attenuate bone metastasis, suggesting the potential role of ADGRG1 in facilitating the BoM process. Regarding COL3A1, Wu and Zheng (46) found that COL3A1 is one of the key genes relating to the brain metastasis process in breast cancer. The interaction between COL3A1 from fibroblasts and ADGRG1 from cancer cells might contribute to the BoM process and these findings are worth future validation through either *in vitro* or *in vivo* studies.

Interestingly, through Pearson analysis, we also find a significant correlation between EMP1 and COL3A1 expressions in fibroblasts/osteoblasts among three types of cancer (**Figures 6A**, **7F**, and **8F**), suggesting a potential connection between these two genes. Zeng et al. (47) found upregulated expression of genes encoding collagen fibers (such as COL1A1, COL1A2, COL3A1, etc.) and matrix proteases (such as MMP2, MMP7, MMP11, etc.) in EMP1^high^ ovarian cancer samples. EMP1 is involved in GO functions such as “collagen fibril organization”, “extracellular matrix organization”, and “integrin-mediated cell adhesion”, further confirming the connections between these two genes. In the future, more validation methods should be performed to verify the importance of these EMP1^+^/COL3A1^+^ fibroblasts during the BoM process in more types of cancers.

Due to the lack of BoM scRNAseq data in other cancer types, in this study, we only involved datasets from three types of cancers, which greatly limits the applicability of our findings pan-cancer-wide. In the future, with the availability of more scRNAseq datasets and in-depth research on the metastasis of more cancers, we will optimize our findings and further explore the mechanisms of these fibroblasts in promoting the BoM process.

## Conclusion

Through an integrated analysis of transcriptomics data, we identified a special subgroup of EMP1^+^/COL3A1^+^ fibroblasts that are enriched in breast cancer BoM samples, which might facilitate the BoM process through interacting with tumor cells via COL3A1-ADGRG1 communication. Elevated expression of cytokines including BMP2 and CXCL12 were found in these fibroblasts, which might also promote the BoM process. Furthermore, the existence of these fibroblasts was also confirmed in BoM samples of prostate and renal cancers, suggesting the importance of these cells. Finally, a strong correlation was discovered between EMP1/COL3A1 genes and EMT-featured gene pan-cancer-wide, implying a possible underlying mechanism through an EMT perspective. Our findings might contribute to deciphering the BoM mechanism while providing potential targets for future treatments of tumor metastasis.

## Data availability statement

The original contributions presented in the study are included in the article/**Supplementary Material**. Further inquiries can be directed to the corresponding author.

## Author contributions

HD: Conceptualization, Writing – original draft. HW: Formal analysis, Writing – original draft. YL: Formal analysis, Writing – original draft. YJ: Formal analysis, Writing – review & editing. JW: Writing – review & editing. DD: Conceptualization, Writing – review & editing. SD: Conceptualization, Writing – review & editing.
